# Elevated levels of dehydroepiandrosterone as a potential mechanism of dendritic cell impairment during pregnancy

**DOI:** 10.1186/s12865-014-0065-9

**Published:** 2015-01-31

**Authors:** Elena R Chernykh, Olga Yu Leplina, Marina A Tikhonova, Nataliya V Seledtsova, Tamara V Tyrinova, Nataliya A Khonina, Alexandr A Ostanin, Nataliya M Pasman

**Affiliations:** Laboratory of Cellular Immunotherapy, Research Institute of Fundamental and Clinical Immunology, 14, Yadrintsevskaya St., 630099 Novosibirsk, Russia; Novosibirsk State University, 2, Pirogova St., 630090 Novosibirsk, Russia

**Keywords:** *Dendritic cells*, *Dehydroepiandrosterone sulfate (DHEAS)*, *Pregnancy*, *Tolerogenic activity*

## Abstract

**Background:**

This study aimed to test the hypothesis that immune dysfunction and the increased risk of spontaneous abortion in pregnant women with hyperandrogenia (HA) are caused by the reduced tolerogenic potential of dendritic cells (DCs) that results from elevated levels of dehydroepiandrosterone sulfate (DHEAS).

**Methods:**

The phenotypic and functional properties of monocyte-derived DCs generated from blood monocytes from non-pregnant women, women with a normal pregnancy, or pregnant women with HA, as well as the *in vitro* effects of DHEAS on DCs in healthy pregnant women were investigated.

**Results:**

In a normal pregnancy, DCs were shown to be immature and are characterized by a reduced number of CD83^+^ and CD25^+^ DCs, the ability to stimulate type 2 T cell responses and to induce T cell apoptosis. By contrast, DCs from pregnant women with HA had a mature phenotype, were able to stimulate both type 1 (IFN-γ) and type 2 (IL-4) T cell responses, and were characterized by lower B7-H1 expression and cytotoxic activity against CD8^+^ T cells. The addition of DHEAS to cultures of DCs from healthy pregnant women induced the maturation of DCs and increased their ability to activate type 1 T cell responses.

**Conclusion:**

Our data demonstrated the reduction in the tolerogenic potential of DCs from pregnant women with HA, and revealed new mechanisms involved in the hormonal regulation of DCs mediated by DHEAS.

## Background

The hypothesis that tolerance plays a major role in pregnancy was first proposed by P. B. Medawar more than 50 years ago [1]. Subsequent studies not only confirmed the assumptions of Medawar, but also resulted in significant progress towards understanding the phenomenon of immunological tolerance. It has become evident that despite the anatomical separation of the mother and fetus, the induction of tolerance requires the recognition of fetal antigens by maternal immune cells [2]. Tolerance induction involves several mechanisms, including immunological ignorance [3], T cell apoptosis and anergy [4,5], and immunosuppression mediated by both regulatory cells [6,7] and cytokines [8,9]. Furthermore, a key role for dendritic cells (DCs) in immune tolerance that occurs during pregnancy has been proposed.

DCs are best known as cells that can induce an adaptive immune response [10,11], although DCs were also recently shown to be capable of suppressing immune responses [12]. Initially, this tolerogenic activity was thought to be associated with an immature state of DCs or with the plasmacytoid DC subset [12,13]. However, subsequent studies showed that intermediate and mature DCs can also exhibit tolerogenic properties [14,15], and DC phenotypes can be influenced by various anti-inflammatory and immunosuppressive mediators [16-18]. DCs can acquire tolerogenic features upon exposure to prostaglandin E_2_, vitamin D_3_, HLA-G molecules, IL-21, IL-16, IL-4, thrombopoietin, M-CSF, G-CSF, HGF, VIP (vasoactive intestinal peptide), or TSLP (thymic stromal lymphopoietin), which are present in large amounts in the decidua [3,12,19,20]. Additionally, many apoptotic trophoblast cells enter the circulation during pregnancy, and their engulfment might also induce tolerogenic activity in DCs [21].

The functional activity of DCs has been shown to be controlled by hormones, including glucocorticoids and sex hormones, such as chorionic gonadotropin, estrogens, and progesterone, which can increase the tolerogenic potential of DCs [22-27]. Therefore, it was suggested that hormones can modulate DCs and that the microenvironment represents a key factor that can trigger and regulate immunological tolerance [28]. Accordingly, dysfunction in hormone secretion pathways can significantly affect the tolerogenic potential of DCs.

Previously, we showed that pregnant women with elevated levels of DHEAS, which results from minor forms of adrenal hyperandrogenia (HA), exhibit immune abnormalities. In particular, they show a reduced number of CD4^+^CD25^+^ regulatory T cells and an increased number of activated NK cells compared to women with a normal pregnancy [29]. Because similar immune abnormalities have been described in women with recurrent miscarriages [30,31], we suggested that immune dysfunction might be the cause of the increased abortion rate in pregnant women with elevated levels of DHEAS [32,33]. The stimulatory effect of DHEAS on the immune system that results in the activation of natural killer cells (NK cells) and Th1 cells is well-known [34]. We have also shown that DHEAS can induce the *in vitro* maturation and Th1-stimulating activity of DCs [35]. Therefore, we suggest that immune dysfunctions in pregnant women with increased levels of DHEAS could result from impaired immunological tolerance, and the reduction in the tolerogenic potential of DCs might be caused by elevated concentrations of DHEAS.

To test this hypothesis, we studied the phenotypic and functional properties of monocyte-derived DCs in non-pregnant women, women with a normal pregnancy, and pregnant women with elevated levels of DHEAS. We also assessed *in vitro* the effect of DHEAS on DCs in healthy pregnant women.

## Methods

### Patients

This study included three groups of women (Table 1). The control group included 42 fertile non-pregnant women (who had at least one successful pregnancy and no previous abortions) whose menstrual cycles were regular (mean age 30.2 ± 0.2 years). All women in the control group had normal serum levels of DHEAS. Study group I included 66 healthy pregnant women who did not have any infectious or endocrine diseases, or clinical signs of hyperandrogenism, and who exhibited normal serum levels of DHEAS (<1.8 mg/ml). Study group II included 44 pregnant women with elevated levels of DHEAS (>1.8 mg/ml) that could be attributed to minor forms of adrenal hyperandrogenia and were not associated with polycystic ovaries. All pregnant women were under 22 weeks of gestation. The groups of pregnant women were comparable in age (26.1 ± 0.1 and 26.9 ± 0.1 years, respectively) and gestation period (14.2 ± 0.2 and 15.1 ± 0.1 weeks, respectively). All patients participating in this study had no acute exacerbations of chronic diseases, acute infections, or other endocrine disorders. All individuals provided written informed consent to participate in this study that was approved by the local ethical committee of the Institute of Fundamental and Clinical Immunology.

**Table 1:** **Characteristics of groups of patients**

**Parameters**	**Groups**		
	**Non-pregnant women (n = 42)**	**Pregnant women with normal levels of DHEAS < 1.8 (n = 66)**	**Pregnant women with elevated levels of DHEAS >1.8 (n = 44)**
Age	27.2 ± 0.2	26.1 ± 0.1	26.9 ± 0.1
Age at menarche	13.2 ± 0.1	13.1 ± 0.1	14.3 ± 0.1
Spontaneous abortion	0	0	16%
Gestation period	-	14.2 ± 0.2	15.1 ± 0.1
DHEAS (μg/ml)	0.58 ± 0.03	0.61 ± 0.04	4.3 ± 0.23
	0.2–1.8	0.5–1.8	2.0–11.2
Testosterone (nmol/l)	0.45–3.75	0.9–5.4	1.0–7.3
Clinical signs of			
- hyperandrogenism	0	0	86%
- menstrual irregularities	0	0	50%
- hair growth in androgen-			
Dependent areas	0	0	43%
- acne	0	0	64%
- oily seborrhea	0	0	38%

### Generation of DCs

Peripheral blood mononuclear cells (MNCs) were obtained by density gradient centrifugation (Ficoll-Paque, Sigma–Aldrich) of heparinized whole blood samples. DCs were generated by culturing the plastic-adherent MNC fraction in 6-well plates (Nunclon, Denmark) in RPMI-1640 medium (Sigma–Aldrich) supplemented with 0.3 mg/ml L-glutamine, 5 mM HEPES buffer, 100 μg/ml gentamicin, and 5% fetal calf serum (FCS, Sigma–Aldrich) in the presence of recombinant human GM-CSF (40 ng/ml, Sigma–Aldrich) and rIFN-α (Roferon-A, 1000 U/ml, Roche, Switzerland) for 4 days at 37°С in a 5% СО_2_ atmosphere (IFNα-DCs). The resulting DCs were then stimulated with 10 μg/ml lipopolysaccharide (LPS *E. coli* 0114: B4, Sigma–Aldrich) as a maturation stimulus for an additional 24 h. In some experiments, DHEAS (Sigma–Aldrich, 10^−6^ М) was added to the DC culture along with LPS. The viability of IFN-DCs, as determined by Trypan blue exclusion, was at least 93–95% in all cases.

### Flow cytometric analysis

Flow cytometry was performed using FACS Calibur and CellQuest software (BD Becton Dickinson). DC phenotypes were determined following direct single- or two-color staining with FITC-, PE- or PerCP-conjugated mAb specific for CD83, CD25, HLA-DR, CD14, or B7-H1 (BD PharMingen). In each experiment, isotype-matched control mAbs were included to measure non-specific background staining.

### Analysis of intracellular expression and production of cytokines

The capacity of DCs to activate T cell cytokine production was tested in allogeneic mixed lymphocyte cultures (MLCs). Freshly isolated monocyte-depleted allogeneic MNCs (1 × 10^5^ per well) were cultured in complete culture medium supplemented with 10% FCS with or without DCs (1 × 10^4^ per well) in 96-well plates for 72 h. For the last 18 h of culture, 10 μg/ml Brefeldin A (Sigma–Aldrich) was added. Cells were harvested, washed, and incubated with APC-conjugated anti-CD3 mAb (BD Pharmingen) at room temperature for 15 min. Cells were then permeabilized with 0.2% Tween-20 (Sigma–Aldrich), stained with FITC-labeled anti-IFN-γ or PE-labeled anti-IL-4 mAb (BD Pharmingen), and analyzed by flow cytometry.

To measure IFN-γ and IL-4 secretion by T cells, after 5 days supernatants of co-cultured MNCs and allogenic IFN-DCs (at a MNC-to-DC ratio of 10:1) were collected and assayed using IFN-γ and IL-4 ELISA kits according to the manufacturer’s instructions (Vector-Best, Russia).

### Analysis of apoptosis

To analyze the apoptosis of CD3^+^CD4^+^ and CD3^+^CD8^+^ Т cells, IFN-DCs were co-cultured with 1 × 10^5^ allogenic MNCs from healthy donors in 96-well round-bottom plates at a DC-to-MNC ratio of 1:10. After 3 days of incubation, cells were harvested, washed, and incubated with APC-conjugated anti-CD3 and PerCP-conjugated anti-CD4 Abs (BD Pharmingen) at room temperature for 15 min. The percentage of apoptotic cells was determined using a FITC-conjugated Annexin V Apoptosis Detection Kit I according to the manufacturer’s recommendations (BD Pharmingen). Apoptotic cells were identified by the binding of FITC-conjugated Annexin V and PI within the CD3^+^CD4^+^ or CD3^+^CD8^+^ (CD4^−^) gates. Data were expressed as the percentage of positive cells among total CD3^+^CD4^+^ and CD3^+^CD8^+^ Т cells. At least 10,000 events were gathered from each sample.

### Statistical analysis

Statistical analysis was performed using Statistica 6.0 software for Windows (StatSoft Inc.). Data were expressed as means ± SE. The nonparametric Mann–Whitney U test was used to determine statistically significant differences. A threshold for significance of p < 0.05 was used.

## Results

**Figure 1:**
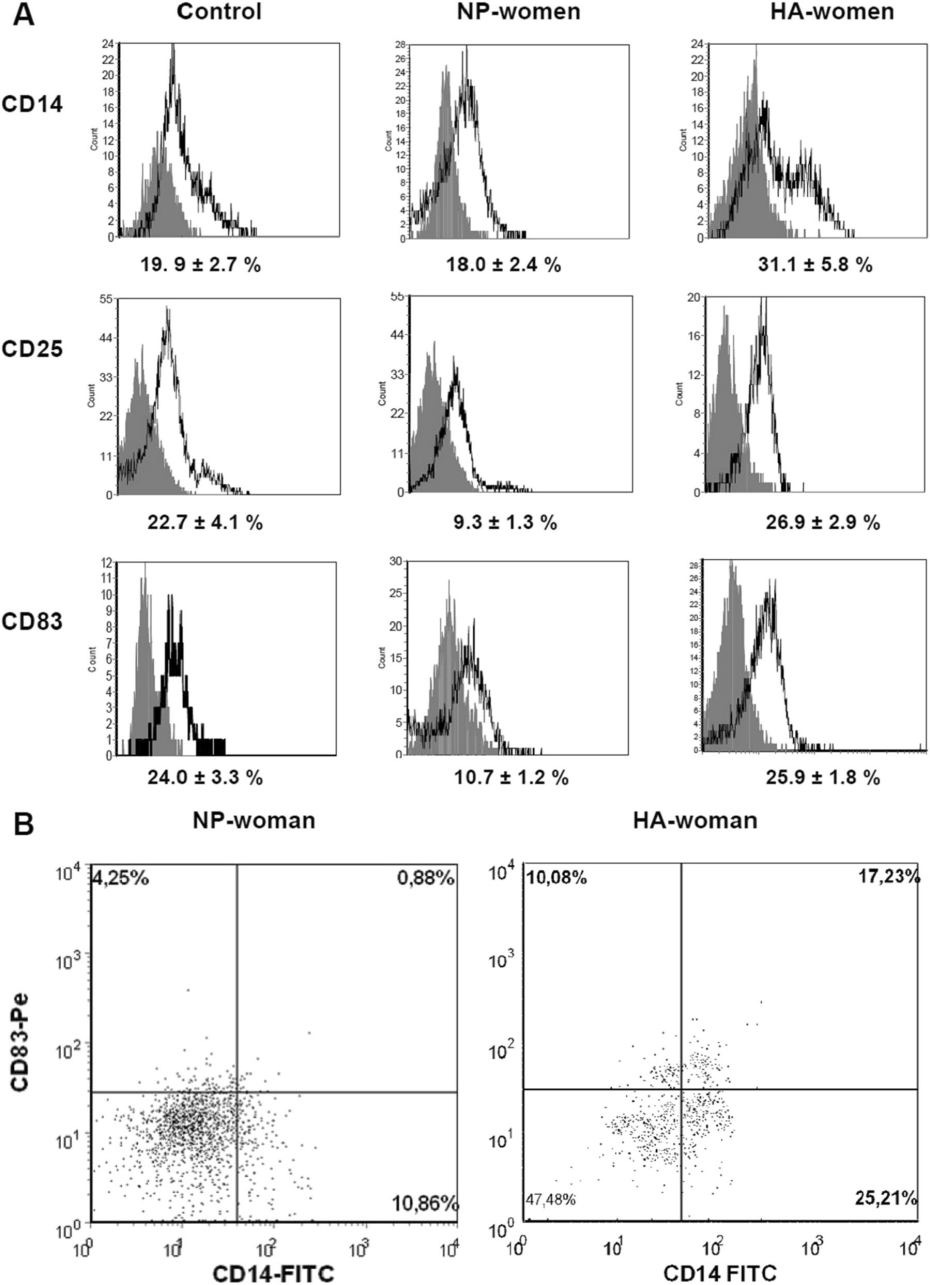
**Phenotypic characteristics of IFN-α-DCs in normal pregnant women and pregnant women with hyperandrogenia.** IFN-α-DCs were analyzed by flow cytometry for the expression of DC-related surface antigens in non-pregnant reproductive aged women (control group), normal pregnant women (NP-women), and pregnant women with hyperandrogenia (HA-women). **(A)** Histograms represent the expression of the indicated molecules (bold-line histograms) and the corresponding isotype controls (grey-filled histograms) in individual experiments. The relative percentage of positive cells (%) among the DCs are presented as means (± SE). **(B)** Gated HLA-DR^+^ IFN-α-DCs were analyzed for CD14 and CD83 staining. Representative stainings of IFN-α-DCs from NP-women (left, dot plots) and HA-women (right, dot plots) are shown.

Because the functional activity of DCs largely depends on their maturity, we compared surface marker expression of DCs generated in normal pregnant women (NP-DCs) and pregnant women with hyperandrogenia (HA-DCs). DCs of non-pregnant fertile women were used as a control (control DCs). As shown in Figure 1, a two-fold lower percentage of CD83^+^ cells (a marker of mature monocyte-derived DCs) and CD25^+^ cells (a marker of activated mature DCs) were generated from normal pregnant women than from non-pregnant controls (р_U_ < 0.01). Meanwhile, HA-DCs were not found to have a reduced percentage of CD83^+^ and CD25^+^ cells as compared to control DCs. Accordingly, the rate of CD83^+^ and CD25^+^ cells in the HA-DC population was two-fold higher than in NP-DCs. The percentage of CD14^+^ cells (a monocyte/macrophage marker) in HA-DC cultures was also significantly higher than in NP-DCs. To explain the cause of the simultaneous increase in CD83^+^ and CD14^+^ cells, we characterized the co-expression of these molecules by DCs. In cultures of NP-DCs, about half of CD83^+^ cells co-expressed CD14 (4.3% ± 0.9%) on their surface. The proportion of CD14^+^CD83^+^ cells in HA-DCs was three times higher than that of women with a normal pregnancy (up to 14.0% ± 2.5%, p_U_ < 0.01), and this explained the observed simultaneous increase in both CD14^+^ and CD83^+^ cells in the HA-DC populations.

To assess whether the impaired maturation/activation state of DCs could affect their functional activity, we further investigated the ability of DCs to stimulate T cell production of IFN-γ and IL-4 in a MLC. DCs from non-pregnant controls (Table 2) stimulated a five-fold increase in the number of CD3^+^IFN-γ^+^ T cells, but negligibly influenced the number of CD3^+^IL-4^+^ T cells. Similar data were also obtained from an analysis of IFN-γ and IL-4 secretion. The concentration of IFN-γ in the supernatant of MLC, stimulated by DCs from non-pregnant women, was significantly higher than that in cultures without DCs. However, DCs did not significantly stimulate IL-4 secretion.

**Table 2:** **T1/T2-stimulatory activity of IFN-α-DCs**

	**Culture conditions**	**Intracellular expression and production of the cytokines IFN-γ and IL-4**					
		**Control group (n = 10)**		**NP women (n = 8)**		**HA women (n = 8)**	
		**Value**	**SI**	**Value**	**SI**	**Value**	**SI**
CD3^+^ IFN-γ^+^ (%)	0	1.5 ± 0.2		1.8 ± 0.5		1.8 ± 0.5	
	+ DCs	5.9 ± 0.6	5.2 ± 0.9	1.5 ± 0.27**	1.1 ± 0.2**	2.5 ± 0.3**^#^	2.0 ± 0.4**^#^
CD3^+^ IL-4^+^ (%)	0	1.7 ± 0.1		2.6 ± 0.4		3.3 ± 0.5	
	+ DCs	2.1 ± 0.4	1.3 ± 0.2	8.6 ± 1.0**	3.5 ± 0.4**	7.4 ± 1.0**	2.4 ± 0.5*
IFN-γ (pg/ml)	0	18.2 ± 5.7		16.5 ± 2.8		16 ± 3.0	
	+ DCs	353 ± 41	22.2 ± 3.1	63.4 ± 27.8**	3.4 ± 0.9**	218 ± 69* ^#^	13.5 ± 3.1*^#^
IL-4 (pg/ml)	0	1.0 ± 0.08		1.0 ± 0.09		1.0 ± 0.12	
	+ DCs	2.0 ± 1.0	2.0 ± 1.0	4.6 ± 2.2	4.6 ± 2.2	5.4 ± 2.0*	5.4 ± 2.0*

Donor MNCs were co-cultured with allogenic IFN-α-DCs from non-pregnant women (control), normal pregnant women (NP), or hyperandrogenia pregnant women (HA) for 72 h at an MNC-to-DC ratio of 10:1. For the final 18 h of culture, 10 μg/ml Brefeldin A was added. Intracellular expression of IFN-γ and IL-4 was detected within the CD3^+^ gate by flow cytometry. To determine the levels of cytokine production by MNCs, cell-free supernatants were collected after 5 days of co-culturing donor MNCs and allogenic IFN-α-DCs from the control group, NP-women, or HA-women (at a MNC-to-DC ratio of 10:1) and were assessed for production of IL-4 and IFN-γ using specific ELISA kits. Data are presented as mean (± SE) cytokine concentrations (pg/ml) or the percentage of CD3^+^ cells from the indicated number of independent experiments. SI indicates the stimulation index, which was calculated as the ratio of cytokine concentrations (pg/ml) or intracellular cytokine expression (%) by MNCs in the presence or absence of DCs. *, p_U_ < 0.05, **, p_U_ < 0.01, compared to the control women group; ^#^, p_U_ < 0.05, compared to the NP-women group. Significant differences were detected using the Mann–Whitney U test.

Compared to control DCs, NP-DCs did not induce an increased number of CD3^+^IFN-γ^+^ T cells and stimulated less secretion of IFN-γ, but did induce almost a three-fold increase in CD3^+^IL-4^+^ T cells and moderately enhanced IL-4 production. HA-DCs also stimulated the increase in CD3^+^IFN-γ^+^ T cells and secretion of IFN-γ, although their stimulatory effect was significantly lower than that of control DCs. Additionally, HA-DCs retained the ability to stimulate T2-responses, in particular to induce CD3^+^IL-4^+^ T cells and increase IL-4 secretion.

To clarify whether the enhanced capacity of HA-DCs to stimulate IFN-γ production by T cells could be attributed to increased level of DHEAS, we studied the direct effects of the hormone on the T1-stimulated activity of NP-DCs. Treatment of NP-DCs with DHEAS induced pronounced Th1-stimulatory activity. Levels of IFN-γ in MLCs induced by DHEAS-modified NP-DCs were more than two-fold higher than that in the MLC, which were induced by intact NP-DCs (344 ± 160 vs. 144 ± 90 pg/ml; P_U_ < 0.05). Therefore, our findings suggest that DHEAS *in vitro* has a pronounced up-regulating effect on Th1-stimulating capacity of NP-DCs.

Notably, DC tolerogenic activity might be associated with both the induction of Th2-responses and the ability of T cells to promote the apoptosis/anergy of cytotoxic T cells. Indeed, we recently found that donor IFN-α-DCs can induce T cell apoptosis via the PD-1/PD-L1 pathway, and enhanced expression of PD-L1 (B7-H1) on DCs could significantly increase their pro-apoptogenic activity in immunopathology [36,37]. Therefore, we compared the expression of B7-H1 and assessed DC cytotoxic activity in normal pregnant women and pregnant women with HA.

**Figure 2:**
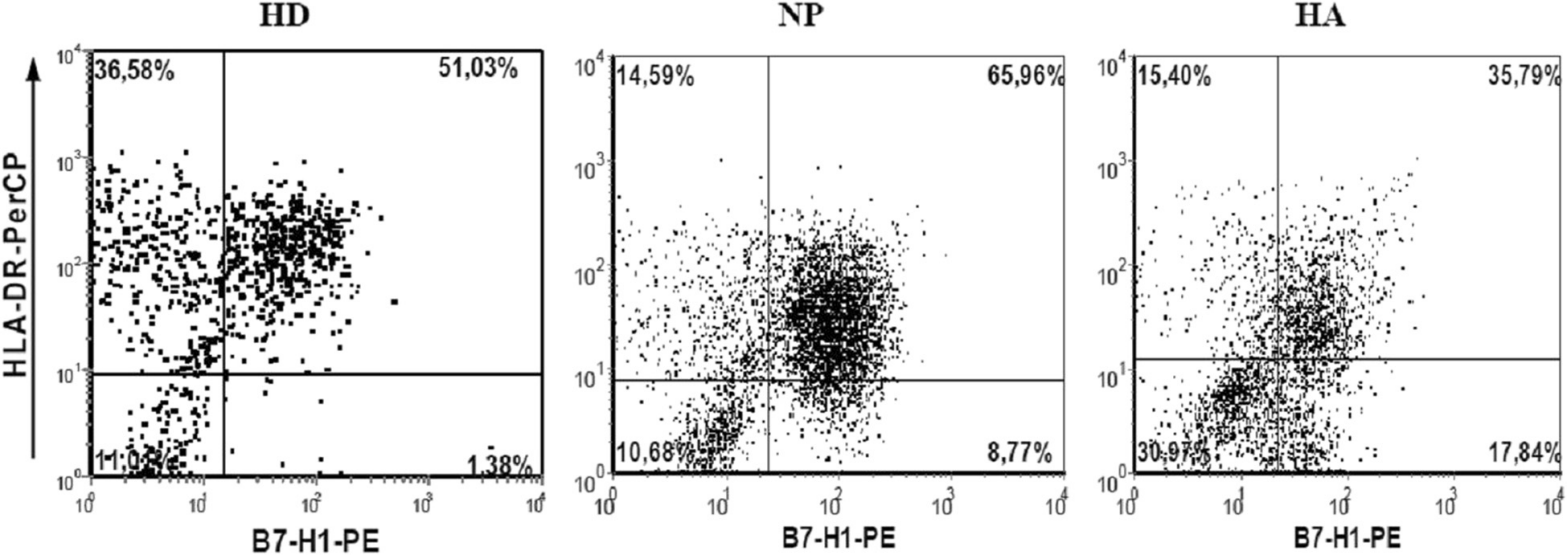
**Expression of B7-H1 molecules by IFN-α-DCs from healthy donors (HD), normal pregnant (NP) women, and pregnant women with hyperandrogenia (HA).** Data are presented as means (± SE) percentage of cells positive for B7-H1 staining among all IFN-α-DCs expressing HLA-DR. *, p_U_ < 0.05 indicates a significant difference compared to healthy donors (n = 12); #, p_U_ < 0.05 indicates a significant difference between groups of NP-women (n = 6) and HA-women (n = 8); comparisons were made using the Mann–Whitney U test.

**Figure 3:**
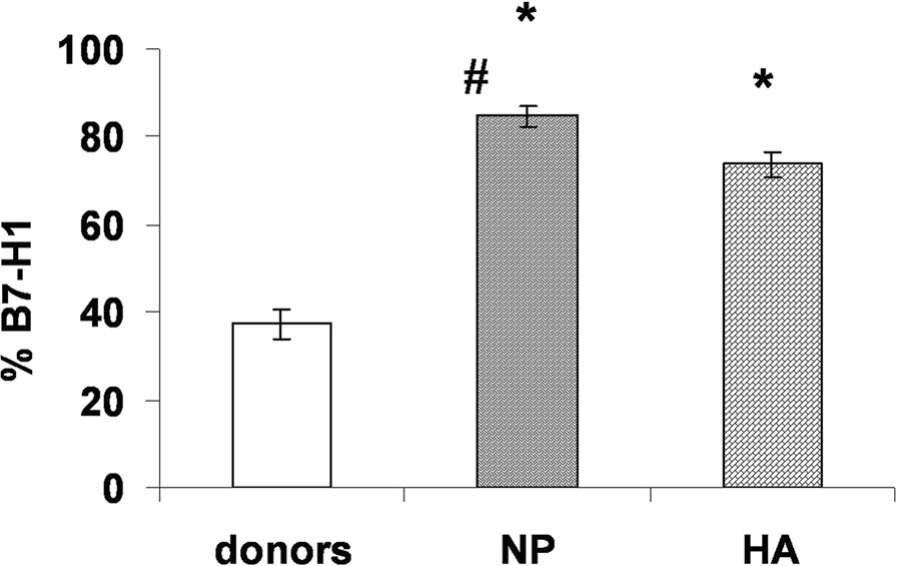
**Expression of B7-H1 by IFN-α-DCs from healthy donors or pregnant women.** Representative data from experiments performed with IFN-α-DCs from healthy donors (HD, left), normal pregnant women (NP, middle), or pregnant women with hyperandrogenia (HA, right) are shown.

The percentage of B7-H1^+^ cells in the HLA-DR^+^ subset of control DCs (Figure 2) varied from 20% to 54%, and the average was 37.3% ± 3.4%. In normal pregnancy, the number of B7-H1^+^ DCs was significantly higher and reached an average of 85% ± 2.6%. The number of B7-H1^+^ cells in HA-DC cultures was significantly lower than in NP-DC cultures (73.7% ± 2.7% vs. 85% ± 2.6%, р_U_ = 0.027). Representative histograms of DC expression of HLA-DR and B7-H1 in women with a normal pregnancy or a pregnancy with HA are shown in Figure 3.

**Figure 4:**
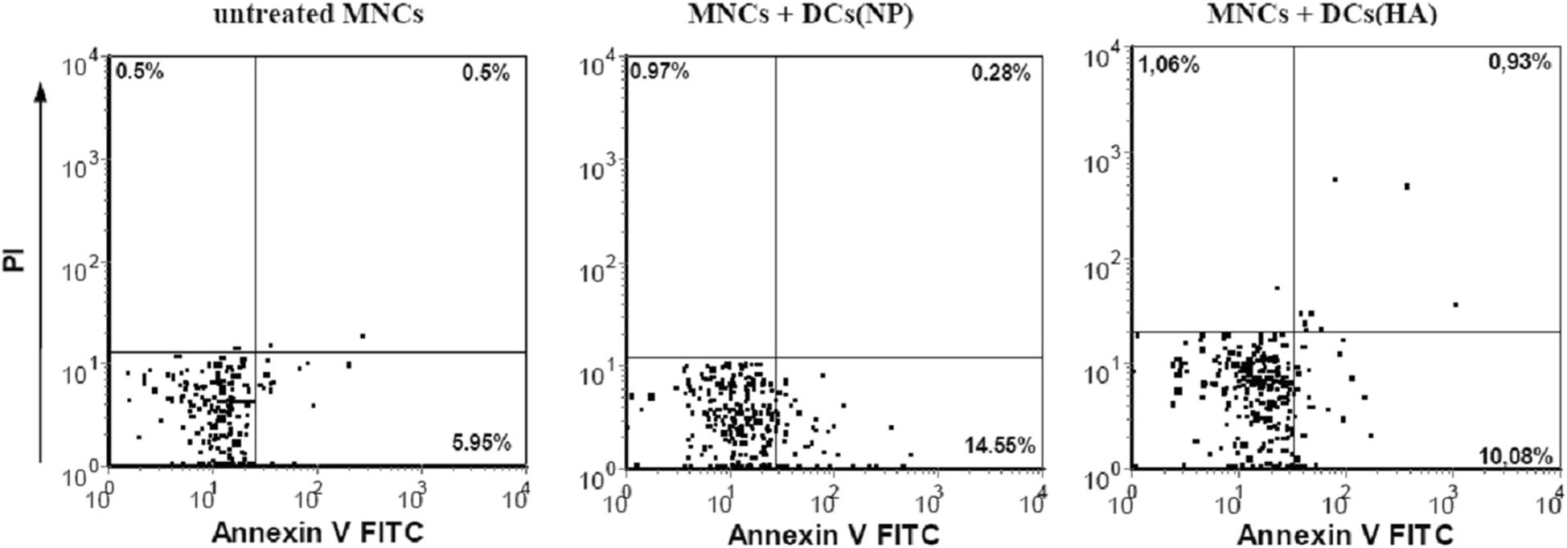
**T cells undergo enhanced apoptotic cell death when cultured with IFN-α-DCs from pregnant HA** ^**+**^ **women.** IFN-α-DCs were co-cultured with 1 × 10^5^ allogenic MNCs from healthy donors at a 1:10 ratio. After 3 days, apoptosis was evaluated by flow cytometry. Gated CD3^+^CD4^+^ T cells were analyzed for Annexin V and PI double positivity. Representative cases of untreated MNCs (left), MNCs co-cultured with IFN-DCs from normal pregnant (NP) women (middle), and MNCs co-cultured with IFN-α-DCs from pregnant women with (HA) hyperandrogenia (right) are shown.

To analyze DC cytotoxic activity of NP-DCs and HA-DCs, we studied the amount of CD4^+^ and CD8^+^ T-cell apoptosis in MLC that was induced by these DCs (Table 3 and Figure 4). NP-DCs and HA-DCs had similar pro-apoptogenic activity against CD3^+^CD4^+^ T cells. The number of apoptotic (Annexin V^+^/PI^−^) CD3^+^CD4^+^ T cells in MLC that were induced by DCs from pregnant women in both groups significantly exceeded the number of apoptotic cells in the controls (monocyte-depleted MNC cultures without DCs), but did not differ from each other. However, the percentage of apoptotic (Annexin V^+^/PI^−^) CD3^+^CD8^+^ T cells in MLC that were induced by NP-DCs was significantly higher compared to MLC that were induced by HA-DCs.

**Table 3:** **IFN-α-DC induced apoptosis of CD3** ^**+**^ **CD4** ^**+**^ **and CD3** ^**+**^ **CD8** ^**+**^ **Т cells**

**Annexin V** ^**+**^ **/PI** ^**−**^ **(%)**	**Untreated MNCs, n = 8**	**MNCs + DCs (NP), n = 6**	**MNCs + DCs (HA), n = 8**
CD3^+^CD4^+^	0.19 ± 0.05	4.7 ± 1.0*	5.3 ± 1.2*
Т cells	0.25	3.6	3.92
CD3^+^CD8^+^	0.17 ± 0.04	15.02 ± 0.3*	11.19 ± 0.9*^#^
Т cells	0.13	15.0	11.23

Untreated MNCs from healthy donors (control) and MNCs co-cultured with IFNα-DCs from normal pregnant (NP) women or pregnant women with hyperandrogenia (HA) at a MNC-to-DC ratio of 10:1 for 3 days were analyzed for Annexin V/PI double positivity by flow cytometry. Data are presented as mean (± SE) percentages and medians of apoptotic Annexin V^+^/PI^−^ cells within the CD3^+^CD4^+^ or CD3^+^CD8^+^ (CD4^−^) gates. *, p_U_ <0.05, compared to the control group; ^#^, p_U_ < 0.01, a significant difference between the NP pregnant women and HA pregnant women groups; differences were detected using the Mann–Whitney U test.

## Discussion

DCs represent unique antigen-presenting cells that are capable of inducing both an immune response and a state of immunological tolerance [10]. The presence of DCs in the decidua, where direct interactions between fetal and maternal cells occurs, makes these cells candidates to be key regulators of immune tolerance in pregnancy [38,39]. Nevertheless, DCs in human pregnancy are not well studied and findings are often contradictory.

In this paper, we demonstrated for the first time that DHEAS, whether elevated during pregnancy *in vivo* or added *in vitro*, results in the induction of DC maturation and type 1 T cell stimulatory activity and reduces the apoptosis-inducing activity of monocyte-derived DCs. Monocytes represent a major source of DC precursors and can differentiate into DCs upon exposure to GM-CSF in combination with IL-4 (IL-4-DCs) or IFN-α (IFN-α-DCs) [40,41]. Because DHEAS has been reported to inhibit the secretion of IL-4 [42], we chose a DC generation protocol using GM-CSF and IFN-α. The choice of this protocol was also motivated by the fact that unlike the IL-4-DCs that predominantly activate Th1-responses, IFN-α-DCs can activate both Th1- and Th2-responses [43,44] and possess more pronounced cytotoxic activity [45]. These attributes provide the opportunity to assess the effects of DHEAS on the Th1- and Th2-stimulating activity and cytotoxic effects of DCs.

Our data have shown that in a healthy pregnancy, monocyte-derived IFN-α-DCs possess “tolerogenic” potential, as evidenced by a less mature phenotype and increased priming of type 2 (IL-4) T cell responses compared to DCs from non-pregnant women. Whereas DCs from non-pregnant women induce activated T cell production of IFN-γ, DCs from women with a normal pregnancy predominantly stimulated the production of IL-4. Our data are consistent with the findings of other groups, which demonstrate that the immature phenotype of myeloid DCs, reduced expression of co-stimulatory molecules (CD86 and CD80), and the expression of immunoglobulin-like transcript 3 (ILT-3) were involved in the induction of tolerance and the higher T2-stimulatory activity of decidual DCs in healthy pregnant women [46-48]. Regarding the properties of circulating DCs, Della Bella *et al.* showed that despite the higher expression of co-stimulatory molecules and the secretion of pro-inflammatory and regulatory cytokines, DCs in a healthy pregnancy were characterized by a low HLA-DR expression, which indicates the “incomplete” activation of these DCs. Moreover, when blood serum from pregnant women was added to control DCs, it induced a DC phenotype associated with low allo-stimulatory activity [49]. For monocyte-derived DCs, Bachy *et al.* showed that DCs generated by culturing cells from a normal pregnancy in the presence of GM-CSF and IL-4 were phenotypically less mature and characterized by reduced IL-12 and increased IL-10 secretion [50]. However, these authors did not investigate the capacity of these DCs to activate T1 and T2 immune responses.

The second important finding of this study is that DCs from women with elevated levels of DHEAS exhibit no signs of immaturity and, along with a capacity to stimulate the T cell secretion of IL-4, can also activate the production of IFN-γ. Because DHEAS, when added to DCs derived from women with a normal pregnancy, induced T1-stimulatory activity, we suggest that the capacity of HA-DCs to prime type 1 T cell responses in pregnant women with HA could be explained by the elevated levels of this hormone.

Another interesting aspect of this study is our analysis of DC cytotoxic activity. Previously, we showed that IFN-α-DCs from healthy donors can induce the apoptosis of activated NK cells, and the maturation of IFN-α-DCs upon exposure to DHEAS is associated with the down-regulation of cytotoxic activity [51]. Furthermore, we showed that IFN-α-DCs could induce T cell apoptosis, which is largely mediated through the PD-L1 (B7-H1)/PD-1 signaling pathway [47]. As B7-H1 expression on IFN-α-DCs declines along with DC maturation [52], we suggest that DC maturation induced by DHEAS could decrease DC cytotoxic potential against T cells. Indeed, our study showed that in a normal pregnancy DCs were characterized by increased expression of B7-H1 compared to control DCs. Furthermore, in pregnant women with elevated levels of DHEAS, the number of B7-H1^+^ DCs was significantly lower than in a normal pregnancy. Moreover, the reduced expression of B7-H1 was associated with lower DC cytotoxicity against CD3^+^CD8^+^ T cells. Overall, these findings suggest that in pregnant women with elevated levels of DHEAS, the tolerogenic properties of DCs were significantly reduced, which could contribute to impaired “immunosuppressive” re-arrangements of the immune system in HA.

The reduced tolerogenic potential of DCs has been proposed as one of the causative factors of abnormal pregnancy in recurrent pregnancy loss [39] and pre-eclampsia [53]. However, impaired DC functions in pregnant women with increased levels of DHEAS are described herein for the first time. Our findings shed new light on immune–endocrine interactions and their importance in normal and complicated pregnancies.

## Conclusions

We report that in healthy pregnancy IFN-α-induced monocyte-derived DCs are characterized by the immature phenotype, the potent ability to stimulate type 2 T cell responses and to induce T cell apoptosis. In contrast, DCs from pregnant women with hyperandrogenia have a mature phenotype, are able to stimulate both type 1 (IFN-γ) and type 2 (IL-4) T cell responses, and differ by lower apoptosis-inducing activity. In addition, DHEAS, when added in vitro to DCs from healthy pregnants, induces the maturation of DCs and increases their ability to activate type 1 T cell responses. This indicates a reduced tolerogenic potential of DCs from pregnant women with HA and reveals a new mechanism involved in the hormonal regulation of DCs mediated by DHEAS.
